# *Plasmodium malariae* Prevalence and *csp* Gene Diversity, Kenya, 2014 and 2015

**DOI:** 10.3201/eid2304.161245

**Published:** 2017-04

**Authors:** Eugenia Lo, Kristie Nguyen, Jennifer Nguyen, Elizabeth Hemming-Schroeder, Jiaobao Xu, Harrisone Etemesi, Andrew Githeko, Guiyun Yan

**Affiliations:** University of California Irvine, Irvine, California, USA (E. Lo, K. Nguyen, J. Nguyen, E. Hemming-Schroeder, G. Yan);; Southern Medical University, Guangzhou, China (J. Xu);; Kenya Medical Research Institute, Kisumu, Kenya (H. Etemesi, A. Githeko)

**Keywords:** *Plasmodium malariae*, nested PCR, parasite gene copy number, microscopy, diagnostic sensitivity, circumsporozoite protein diversity, csp, co-infections, parasites, malaria, Kenya, Africa

## Abstract

In Africa, control programs that target primarily *Plasmodium falciparum* are inadequate for eliminating malaria. To learn more about prevalence and genetic variability of *P. malariae* in Africa, we examined blood samples from 663 asymptomatic and 245 symptomatic persons from western Kenya during June–August of 2014 and 2015. *P. malariae* accounted for 5.3% (35/663) of asymptomatic infections and 3.3% (8/245) of clinical cases. Among asymptomatic persons, 71% (32/45) of *P. malariae* infections detected by PCR were undetected by microscopy. The low sensitivity of microscopy probably results from the significantly lower parasitemia of *P. malariae.* Analyses of *P. malariae* circumsporozoite protein gene sequences revealed high genetic diversity among *P. malariae* in Africa, but no clear differentiation among geographic populations was observed. Our findings suggest that *P. malariae* should be included in the malaria elimination strategy in Africa and highlight the need for sensitive and field-applicable methods to identify *P. malariae* in malaria-endemic areas.

Over the past decade, malaria control strategies in Africa have reduced the number of malaria cases and deaths. Nevertheless, non–*Plasmodium falciparum* malaria still presents a major challenge for malaria elimination (*1**,**2*). Global malaria elimination programs focus primarily on *P. falciparum*. Recent research efforts and control programs have drawn resources to *P. vivax* malaria. By contrast, *P. malariae* and *P. ovale* receive little attention, and malaria caused by these organisms is among the most neglected tropical diseases (*3*). In those rural areas of Africa where malaria is most common, affordable diagnostic tools are rapid diagnostic tests and microscopy, but they are not effective for detecting these 2 species, mainly because parasitemia with these species is low (*4**–**6*). As a result, *P. malariae* and *P. ovale* infections are often underestimated, and epidemiologic information, such as distribution and prevalence of these species in malaria-endemic areas, is lacking. This knowledge is essential for implementation of specific strategies for monitoring and eliminating all types of malaria where it is endemic to Africa.

Although *P. malariae* infection is often asymptomatic and rarely leads to severe clinical illness or death, this species causes a low-grade chronic infection that persists for decades and is associated with nephropathy and anemia (*7**–**9*). The persistence, as well as submicroscopic features of *P. malariae*, have contributed to intermittent outbreaks of malaria in the Colombian Amazon region (*10*). In addition, *P. malariae* can cause irreversible stage 5 kidney failure (*11*). The prevalence of this species may increase the risk for kidney injuries and impair renal function, particularly in children with no immunity against *P. malariae*. Ample evidence shows peak prevalence for severe and uncomplicated clinical *P. falciparum* malaria among infants and children in sub-Saharan Africa (*12**–**14*). Contrary to this age pattern, patients with *P*. *malariae* infections in Papua, Indonesia, were older (median 22 years of age) than those with non–*P. malariae* infections (e.g., *P. vivax; *median 10 years of age) (*9*). Knowledge of the age patterns of patients with *P. malariae* infection is critical for understanding its epidemiology and developing effective preventative strategies.

Compared with the distribution of *P. falciparum* and *P. vivax,* the distribution of *P. malariae* is relatively sparse and variable. *P. malariae* is endemic to West Africa (*3*), South America (*15*), Asia (*16**,**17*), and the western Pacific region (*18**,**19*). Knowledge of genetic variation among isolates from these geographic areas is still lacking. One study indicated a remarkably low level of sequence diversity at the *msp*1 locus in *P. malariae* from Brazil (*20*). Similarly, the lack of variation at the *dhfr* and *dhps* loci has been shown for *P. malariae* from Asia and the western Pacific region (*21**,**22*). These findings suggested that antimalarial drugs might be imposing selective pressure on the genetic diversity of *P. malariae*. The circumsporozoite protein (*csp*) gene, which is known to be critical for plasmodia sporozoite motility and hepatocyte invasion (*23*), has been shown to be variable in length and is a sequence of the tandemly repeated peptide units in *P. falciparum *(*24**,**25*), *P. vivax *(*26**,**27*), and *P. malariae* isolates from Central Africa (*28*). The vast antigenic variation observed in *P. falciparum* as a result of immune selection pressure can influence the capacity of mosquito transmission and the effectiveness of malaria vaccine (*29*). In this study, we sought to determine the prevalence of infection and age distribution of persons with asymptomatic and symptomatic *P. malariae* infection in western Kenya, the genetic affinity between *P. malariae* isolates from East Africa and other regions, and the level of *csp* gene diversity among *P. malariae* and the significance of this diversity.

Scientific and ethical clearance was given by the institutional scientific and ethical review boards of the Kenya Medical Research Institute and the University of California Irvine. Written informed consent/assent for study participation was obtained from all consenting heads of households, parents/guardians (for minors <18 years of age), and each person who was willing to participate in the study.

## Materials and Methods

### Study Areas and Participants

**Figure 1:**
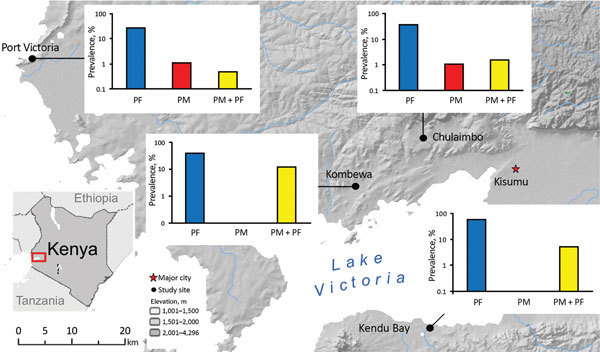
Location of sites in western Kenya for study of *Plasmodium malariae* prevalence and circumsporozoite protein gene diversity, Kenya, 2014 and 2015. Prevalence (logarithmic vertical scales) of *P. falciparum* monoinfections (PF), *P. malariae* monoinfections (PM), and *P. falciparum* and *P. malariae* co-infections (PF+PM) are shown for each study site.

During June–August of 2014 and 2015, blood samples were collected from persons in 4 villages at the Lake Victoria basin (elevation ≈1,000 m) of western Kenya (Figure 1). These villages represent parts of the Lake Victoria area previously shown by nested and quantitative PCR (qPCR) methods to have high, stable rates of malaria transmission and prevalence (10%–40%) among children 5–14 years of age (*30**,**31*). 

Community samples were collected from nonfebrile schoolchildren in 7 public primary schools (70–100 children/school, 2 schools/village except Kombewa). An equal number of boys and girls 6–15 years of age were randomly selected from each school. To determine *P. malariae* prevalence in the adult population, we randomly selected 63 persons (32 male and 31 female) >15 years of age from 18 households in Kombewa. We examined a total of 663 samples from the communities, which provided an estimation of 4% margin of error in parasite prevalence with 0.05 type I error. At the time of sampling, none of these persons exhibited fever or malaria-related symptoms.

Clinical samples were collected from 113 male and 132 female patients, <1 to 76 years of age, in 3 district hospitals. This sample size provided an estimation of 6% margin of error in parasite prevalence with 0.05 type I error. These patients had fever or malaria-related signs or symptoms and were determined to be positive for *Plasmodium* spp. by microscopy at the time of sampling. Thick and thin blood smears were prepared for microscopic examination to determine the *Plasmodium *species, and ≈50 μL blood was blotted onto Whatman 3MM filter (Sigma Aldrich, St. Louis, MO, USA) papers. Filter papers were air dried and stored in zip-sealed plastic bags with silica gel absorbent at room temperature until DNA extraction.

### Microscopy and PCR of *Plasmodium* spp.

We examined slides under microscopes at 100× magnification and counted the number of parasites per 200 leukocytes. A slide was considered negative when no parasites were observed after counting >100 microscopic fields. At the time of sample collection, all slides were read by 2 microscopists. If counts were discordant, the slides were examined by a third microscopist. The density of parasitemia was expressed as the number of asexual parasites per microliter of blood, assuming a leukocyte count of 8,000 cells/μL, according to World Health Organization guidelines.

We extracted parasite DNA from half of a dried blood spot by using the Saponin/Chelex method (*32*). The final extracted volume was 200 μL. For all samples, nested amplification of the 18S rRNA gene region of plasmodia (*P. falciparum, P. vivax, P. malariae*, and* P. ovale*) was used for parasite detection and species identification. As positive controls for all amplifications, we used DNA from *P. falciparum* isolates 7G8 (MR4-MRA-926) and HB3 (MR4-MRA-155), *P. vivax* Pakchong (MR4-MRA-342G) and Nicaragua (MR4-MRA-340G), *P. malariae* (MR4-MRA-179), and *P. ovale* (MR4-MRA-180). As negative controls, we used water and noninfected samples to ensure lack of contamination. Reaction was performed in a Bio-Rad MyCycler thermal cycler according to the published protocol (*33*) (details in Technical Appendix 1).

In addition, the amount of parasite DNA was estimated by using the SYBR Green (Thermo Scientific, Foster City, CA, USA) qPCR detection method with *Plasmodium* species–specific primers that targeted the 18S rRNA genes (*34**,**35*). Reactions were performed in a CFX96 Touch Real-Time PCR Detection System (Bio-Rad, Foster City, CA, USA). To confirm specific amplifications of the target sequence, we performed melting curve analyses for each amplified sample. To measure reproducibility of the cycle threshold (C_t_), we calculated the mean value and standard deviations from triplicates in 2 independent assays. The parasite gene copy number in a sample was quantified according to C_t_ by using the equation (*30*) GCN_sample_ = e^(E × ΔCtsample)^, where GCN stands for gene copy number; ΔCt, the difference in C_t_ between the negative control and the sample; e, exponential function; and E, amplification efficiency (Technical Appendix 1).

### *CSP* Sequencing and Phylogenetic Analyses

Four internal primers were designed specifically on the *P. malariae csp* gene region and used together with the published primers (*28*; Technical Appendix 1 Table) to unambiguously amplify the 3 segments, the N terminal, the central repeat, and the C-terminal regions of the *csp* gene. A total of 37 *P. malariae* isolates were amplified and sequenced. All resulted sequences were verified by comparing them with those in the GenBank database by using BLAST (https://blast.ncbi.nlm.nih.gov/Blast.cgi). Sequences were translated into protein sequences and analyzed together with all *csp* protein sequences available in GenBank of *P. malariae* from East Africa (Kenya and Uganda), West Africa (Cameroon), Central Africa (Côte d’Ivoire), and South America (Venezuela) and of *P. brasilianum* from South America (Brazil and Venezuela). It is noteworthy that although *P. malariae* and *P. brasilianum* coexist in Brazil, no *csp* sequence for *P. malariae* is available. Because of the potential for alignment errors associated with gaps in the nucleotide sequences, we used translated amino acid sequences with unambiguous indels in phylogenetic analyses. Sequence diversity, including measures of evolutionary distances and average pairwise divergence, were estimated and compared among geographic regions (Technical Appendix 1).

### Statistical Analyses

A 1-tailed *t*-test was used to test for the significance of differences in parasite gene copy number between *P. malariae *from symptomatic and asymptomatic patients and between *P. malariae* and *P. falciparum* in co-infected samples. In addition, we calculated the Pearson correlation coefficient (*r*^2^) for parasite gene copy number and age by using R (https://www.r-project.org/).

## Results

### *P. malariae* Prevalence and Patient Age Distribution

Among the 663 samples from asymptomatic persons, *P. malariae* was detected by PCR in 35 (5.3% prevalence). Among these, 29 were mixed infections (with *P. falciparum*) and 6 were *P. malariae* monoinfections (Figure 1; Table 1). *P. malariae* was found to be most prevalent in Kombewa (14.3%, 19/133 cases), followed by Kendu Bay (5.3%, 8/150 cases). Prevalence of *P. falciparum* prevalence was relatively high at these 2 sites (44% and 59%, respectively; Table 1). In Kombewa, 13 of 19 *P. malariae* cases were detected in younger persons (<15 years of age), which was significantly higher than the number of cases detected in older persons (6 cases, p = 0.04; Table 1). Although such a comparison between age groups cannot be made for the other sites, a similar pattern was observed for symptomatic patients.

**Table 1:** Prevalence of *Plasmodium malariae* and *P. falciparum* among asymptomatic persons in the community, Kenya, June–August 2014 and 2015*

Site, patient age, y	No. tested	No. (%) infections			
		Total	*P. malariae*	*P. falciparum*	Mixed†
Kombewa					
<15	63	41 (65.1)	0	28 (44.4)	13 (20.6)
>15	70	35 (50)	2 (2.9)	29 (41.4)	4 (5.7)
Chulaimbo					
<15	190	76 (40)	2 (1.1)	71 (37.4)	3 (1.6)
Kendu Bay					
<15	150	97 (64.7)	0	89 (59.3)	8 (6)
Port Victoria					
<15	190	57 (30)	2 (1.1)	54 (28.4)	1 (0.5)
Total	663	306 (46.2)	6 (0.9)	271 (40.9)	29 (4.4)

*According to nested PCR of the 18S rRNA gene. †*P. malariae* and *P. falciparum*.

Among the 245 samples from symptomatic patients, 8 (3.3%) *P. malariae* cases were detected; 6 were mixed infections with *P. falciparum* and 2 were *P. malariae* monoinfections (Table 2). When the samples were stratified by patient age, all *P. malariae* infections in symptomatic persons were in infants or very young children of <5 years of age (8/135, 5.9% infection rate). Although *P. falciparum* infection was highest among patients >5 to <15 years of age, no *P. malariae* was detected in persons in this and older age groups despite smaller samples in these groups. No significant difference was detected between male and female patients.

**Table 2:** Prevalence of *Plasmodium malariae* and *P. falciparum* among symptomatic persons, Kenya, June–August 2014 and 2015*

Site, patient age, y	No. tested	No. (%) infections			
		Total	*P. malariae*	*P. falciparum*	Mixed†
Chulaimbo					
<5	27	18 (66.7)	2 (7.4)	15 (55.6)	0
>5 to <15	4	3 (75)	0	3 (75)	0
>15	13	3 (23.1)	0	3 (23.1)	0
Kendu Bay					
<5	44	38 (86.4)	0	35 (79.5)	3 (6.8)
>5 to <15	34	31 (91.2)	0	31 (91.2)	0
>15	24	23 (95.8)	0	23 (95.8)	0
Port Victoria					
≤5	64	54 (84.4)	0	51 (79.7)	3 (4.7)
>5 to <15	22	20 (90.9)	0	20 (90.9)	0
>15	13	9 (69.2)	0	9 (69.2)	0
Total					
<5	135	110 (81.5)	2 (1.5)	101 (74.8)	6 (4.4)
>5 to <15	60	54 (90)	0	54 (90)	0
>15	50	35 (70)	0	35 (70)	0

*According to nested PCR of the 18S rRNA gene. †*P. malariae* and *P. falciparum*.

### Comparisons of Diagnostic Approaches and Parasitemia

Compared with microscopy, nested PCR revealed a significantly higher number of *P. malariae* infections in the community (Table 3). All samples that were *P. malariae* positive by microscopy were identified as positive by PCR and qPCR. Across the study sites, nested PCR–based prevalence ranged from 0 to 12.2% (average 4.8%), >2-fold higher than by microscopy (0 to 3.8%, average 1.9%; Table 3). The discrepancy between the 2 methods was also reflected by the difference in *P. falciparum* prevalence; 10% more positive infections were detected by nested PCR than by microscopy. Nevertheless, such a discrepancy was not as substantial as that for *P. malariae*.

**Table 3:** Methods used to diagnose *Plasmodium* infections in asymptomatic populations, Kenya, June–August 2014 and 2015*

Site, method	No. tested	No. (%) infections					
		Total	*P. falciparum *	*P. vivax *	*P. malariae *	*P. ovale *	*P. falciparum/malariae*
Kombewa							
Microscopy	133	54 (41.2)	49 (37.4)	0	0	0	5 (3.8)
PCR	133	70 (53.4)	54 (41.2)	0	0	0	16 (12.2)
Chulaimbo							
Microscopy	190	46 (24.2)	42 (22.1)	0	1 (0.5)	0	3 (1.6)
PCR	190	76 (40.1)	71 (37.4)	0	2 (1.1)	0	3 (1.6)
Kendu Bay							
Microscopy	150	78 (52)	75 (50)	0	0	0	3 (2)
PCR	150	97 (64.6)	89 (59.3)	0	0	0	8 (5.3)
Port Victoria							
Microscopy	190	36 (18.5)	35 (18.5)	0	0	0	1 (0.5)
PCR	190	57 (30)	54 (28.4)	0	2 (1.1)	0	1 (0.5)
All sites							
Microscopy	663	214 (32.4)	201 (30.4)	0	1 (0.2)	0	12 (1.8)
PCR	663	300 (45.4)	268 (40.5)	0	4 (0.6)	0	28 (4.2)

**Figure 2:**
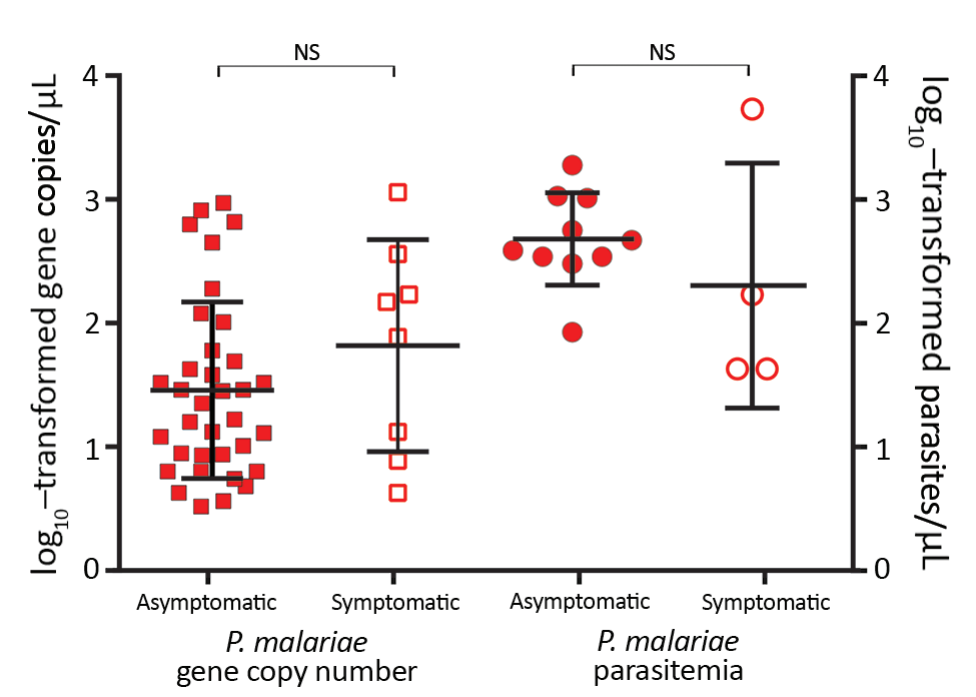
Parasite gene copy numbers (per microliter) detected by SYBR Green (Thermo Scientific, Foster City, CA, USA) quantitative PCR and parasitemia (parasites per microliter) determined by microscopy of *Plasmodium malariae* samples from asymptomatic and symptomatic persons. Median, first quartile, and fourth quartile of the data are shown for each sample category (horizontal lines). No significant difference was observed between asymptomatic and symptomatic persons in terms of *P. malariae* parasite gene copy number and parasitemia. Squares represent samples with gene copy number measured by quantitative PCR; circles, samples with parasitemia estimated by microscopy; closed squares and circles, *P. malariae* samples from asymptomatic persons; open squares and circles, *P. malariae* samples from symptomatic patients. NS, not significant.

Although the number of *P. malariae–*positive clinical samples detected in this study was low, these samples indicated an overlapping range of parasite gene copy number (geometric mean 6.4 × 10^1^/μL, range 4.3 × 10^1^ to 1.2 × 10^3^/μL; Figure 2) with that of the samples from asymptomatic persons (geometric mean 4.8×10^1^/μL, range 0.5 ×10^1^ to 9.4 ×10^2^/μL) without differing significantly (p>0.05). Similar results were observed in the level of *P. malariae* parasitemia, for which samples from symptomatic and asymptomatic persons did not differ significantly (Figure 2). Parasite gene copy number and *P. malariae* parasitemia were significantly positively correlated with each other (*r*^2^ = 0.77, p<0.01; Technical Appendix 1 Figure 1).

**Figure 3:**
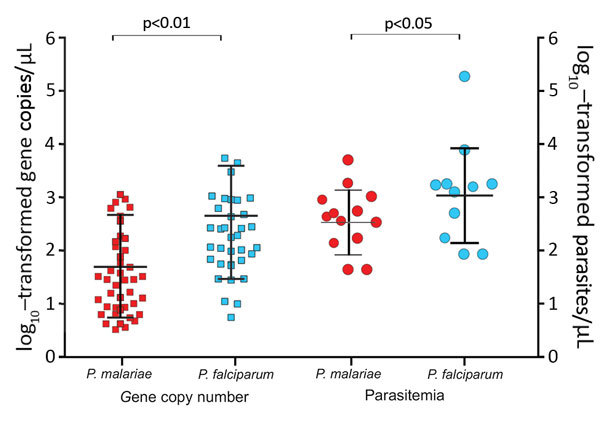
*Plasmodium malariae* and *P. falciparum* parasite gene copy numbers (per microliter) and parasitemia (parasites per microliter) in co-infected samples. Median, first quartile, and fourth quartile of the data are shown for each sample category (horizontal lines). Parasite gene copy number and parasitemia were lower in *P. malariae*–positive than in *P. falciparum*–positive samples. Squares represent samples with gene copy number measured by quantitative PCR; circles, samples with parasitemia estimated by microscopy; red, *P. malariae* samples; blue, *P. falciparum* samples.

Parasite gene copy number and parasitemia for *P. falciparum* were generally higher than those for *P. malariae* (Figure 3, panel A). Among the 35 mixed infections, 28 (80%) gene copy numbers were higher for *P. falciparum* than for *P. malariae* (Technical Appendix 1 Figure 2). Among these samples overall, the amount of *P. falciparum* DNA (geometric mean 1.6 × 10^2^/μL, range 1 × 10^1^ to 5.5× 10^3^/μL) was significantly higher than the amount of *P. malariae* DNA (geometric mean 4.7 × 10^1^/μL, range 0.4 × 10^1^ to 1.1 × 10^3^/μL; p = 0.003), consistent with the difference in parasitemia according to microscopy (*P. malariae* geometric mean 3.2 × 10^2^ parasites/μL vs. *P. falciparum* geometric mean 1.1 × 10^3^ parasites/μL; Technical Appendix 1 Figure 2).

When all *P. malariae* samples were pooled, the parasite gene copy number did not correlate significantly with patient age (*r*^2^ = 0.07; Technical Appendix 1 Figure 3). Neither *P. malariae* prevalence rate nor parasite gene copy number differed significantly according to patient sex. 

### Genetic Relatedness and *csp* Divergence of *P. malariae*

The *csp* alignment comprised 530 aa, of which 34 (6.4%) were polymorphic among the studied parasites of different taxa (Technical Appendix 2). To avoid polymorphism caused by PCR error, we sequenced each isolate at least twice in both directions. Substantial length variation was observed in the central repeat region, where the number of NAAG_n _(the repeat codon unit in which n denotes the number of repeats) in *P. malariae *ranged from 49 to 85 units. These tandem repeats could be rapidly evolving through a different mechanism and may influence genetic relationships among the samples. To examine such effect, we constructed phylogenetic trees with 2 sets of data: the entire sequence (530 aa) and partial sequences without the central repeat region (225 aa).

**Figure 4:**
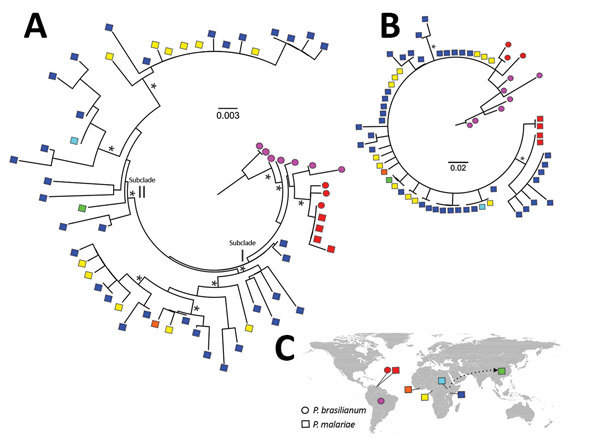
Maximum-likelihood analyses of circumsporozoite protein gene (*csp*) sequences of *Plasmodium malariae* and distribution of the samples. A) Phylogenetic tree based on maximum-likelihood analyses of the entire *csp* amino acid sequences of *P. malariae* isolates from different geographic regions, shown by different colors. Asterisks denote clade with >90% bootstrap support. Sequences of *P. malariae* from Venezuela (red squares) were almost identical to those of *P. brasilianum* (red circles) from the same area. These samples were genetically closely related with *P. brasilianum* from Brazil (violet circles) but distant from *P. malariae *from East and West Africa*.* The samples from Africa were subdivided into 2 subclades, I and II. Subclade I comprised a mix of *P. malariae* isolates from Kenya (dark blue squares), Cameroon (yellow squares), and Côte d’ Ivoire (orange squares). Subclade II comprised a mix of *P. malariae* isolates from Kenya, Cameroon, Uganda (light blue squares), and China (green squares). B) Maximum-likelihood analyses of partial *csp* amino acid sequences without the central repeat region. *P. brasilianum* from Brazil was distant from *P. malariae*, but relationships among the *P. malariae* samples from different geographic areas were not well resolved. C) Locations of samples included in the analyses. Arrow indicates the possible African origin of *P. malariae* from China. Scale bars indicate length of phylogenetic tree. *Bootstrap value >90%.

Maximum-likelihood analyses of the entire *csp* gene showed a clear distinction between isolates from South America and those from the other geographic regions (Figure 4, panel A). *P. brasilianum* and *P. malariae* from Venezuela formed a monophyletic group (bootstrap >95%) closely associated with *P. brasilianum* from Brazil. Sequences of *P. malariae* from Venezuela were almost identical to those of *P. brasilianum* from the same area. Closely related to the clade from South America was a large monophyletic group that contained *P. malariae* from East, Central, and West Africa and from China (bootstrap >90%). The isolates from these regions were divided into 2 subclades: I and II (Figure 4, panel A). Subclade I comprised a mix of *P. malariae* isolates from Kenya, Cameroon, and Côte d’Ivoire. Subclade II comprised a mix of *P. malariae* isolates from Kenya, Cameroon, Uganda, and China. Sequences without the central repeat region indicated consistently the distinctiveness between *P. brasilianum* from Brazil and *P. malariae*, but the *P. malariae* samples from different geographic regions were poorly resolved (Figure 4, panel B). The *P. malariae* isolate from China was nested within the African subclade, suggestive of an African origin (Figure 4, panel C). No clear microgeographic structure was detected, although sample size at the population level was small.

**Figure 5:**
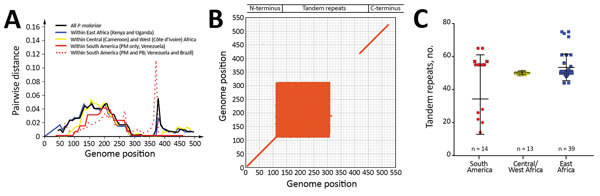
Comparison of circumsporozoite protein (*csp*) gene sequence divergence among *Plasmodium* isolates from different geographic regions. A) Pairwise genetic distance plot of all amino acid positions of the *csp* gene. The matrix-normalized distances based on the standard point accepted mutation (Dayhoff–PAM) model that account for the probability of change from 1 amino acid to another were calculated. Samples were analyzed as a whole and partitioned by geographic regions as indicated by colors. B) Dot plot showing matching scores, a proxy of sequence similarity, between pairwise samples calculated based on the standard Dayhoff–PAM matrix. The greatest mismatch was detected at amino acid positions 110–310, representing the 3′ N terminal through the central repeat regions. C) Variation in the number of tandem repeats in the central region of the *csp* gene. The greatest length variation was observed in the isolates from South America despite the fact that both *P. malariae* (PM) and *P. brasilianum* (PB) were included. *P. malariae* from East Africa was more variable in the number of repeats than isolates from Central/West Africa, despite difference in sample size. Median, first quartile, and fourth quartile of the data are shown for each sample category (horizontal lines). Red represents samples from South America; yellow, Central/West Africa; blue, East Africa. Circles represent *P. brasilianum*; squares, *P. malariae.*

Among the 3 geographic regions, the level of *csp* sequence divergence in *P. malariae* was higher in isolates from East Africa than from West Africa, as reflected by a higher number of polymorphic sites and a greater extent of *csp* length variation despite difference in sample size (Figure 5, panels A and B). These variations were located at the 3′ N terminal through the central repeat region, where the largest degree of mismatch was observed (Figure 5, panel B). To the contrary, the level of sequence polymorphism was lowest in isolates from South America (Figure 5, panel A), but the greatest range of difference in tandem repeat units where remarkable mismatch was observed was toward the end of the central region. Despite the small sample size, the number of tandem repeats was generally lower in *P. brasilianum* than *P. malariae* (Figure 5, panel C). 

## Discussion

In Kenya, areas along the shoreline of Lake Victoria and coastal regions are malaria hot spots, where intense and stable plasmodia transmission occurs throughout the year (*31*). For achieving the ultimate goal of eliminating malaria in Kenya, existing control programs that primarily target *P. falciparum* are inadequate. The use of rapid diagnostic tests or microscopy as first-line diagnostic methods can lead to gross underestimation of the actual prevalence of *P. malariae *(*4**–**6*). Our findings indicated that *P. malariae* accounted for ≈3% of clinical cases and ≈5% of asymptomatic infections in this malaria-endemic region. The prevalence of asymptomatic *P. malariae* infections was comparable to that recently reported for nearby islands of Lake Victoria (1.7%–3.96%) on the basis of PCR (*36**,**37*). These asymptomatic *P. malariae* infections are concerning because they are parasite reservoirs that can sustain long-term transmission. For instance, in the Colombian Amazon region, *P. malariae* was thought to account for <1% of all malaria infections (*38**,**39*); however, a recent study revealed that 43.6% (294/675) of clinical cases were caused by *P. malariae *(*10*) and suggested that these parasites have been circulating in the community undetected. Underestimation or lack of awareness of its occurrence could thus lead to increased transmission. The infectiousness of *P. malariae* for *Anopheles* mosquitoes in malaria-endemic areas remains unclear and merits further investigation.

We found that* P. malariae* infections were more common among infants and children than adults. A similar pattern has been found for Senegal, West Africa, where 91% (265/290 cases) of clinical *P. malariae* cases occurred in children <15 years of age and the mean incidence density was highest for those 5–9 years of age (*3*). These findings indicate that children are vulnerable to *P. malariae* infection and contrast with those reported for Papua, Indonesia, where *P. malariae* infection was higher among older (median 21 years of age) than younger persons (*9*). It is possible that our study sites in western Kenya, as well as in West Africa, are high-transmission areas where *P. falciparum* malaria prevalence can be ≈60% during the rainy season (*30**,**40*). Cumulative exposure to the parasites over time may enable gradual acquisition of immunity in adults. Nevertheless, our community samples were mostly obtained from schoolchildren 6–15 years of age. Underrepresentation of adult populations may underestimate the overall malaria prevalence in the study area. Although young children are more vulnerable to *P. malariae* infections, the level of *P. malariae* parasitemia does not seem to be associated with age. Chronic nephrotic syndromes attributed to *P. malariae* have been reported (*41**,**42*) and shown to be associated with significant illness from anemia in young children (*8**,**9*). However, the lack of hematologic data from our study participants limits further investigation.

Our data indicate that ≈50% of *P. malariae*–positive samples detected by PCR were undetected by microscopy. Such a low sensitivity of microscopy could be attributed to a significantly lower *P. malariae* than *P. falciparum* parasitemia, according to qPCRs. Because most *P. malariae*–positive samples had mixed infections, microscopists could have recorded only the dominant *P. falciparum* and overlooked the sparse *P. malariae* trophozoites. Also, the ring forms of *P. falciparum* and *P. malariae* are morphologically more similar to each other than to *P. vivax* and *P. ovale *(*43*). Misdiagnosis of parasite species by microscopy is possible (*8*). 

In Africa, the standard treatment for *P. malariae* monoinfection is chloroquine, and for *P. falciparum* and mixed plasmodial infections it is artemisinin combination therapy (*31*). The combination treatment regime should cure *P. malariae* infections even in cases of misdiagnosis. However, *P. malariae* increases production of *P. falciparum* gametocytes in mixed infections, and these gametocytes can persist without proper antimalarial treatment or monitoring (*44*). Therefore, we highlight the need for sensitive methods to improve *P. malariae* diagnosis and provide accurate epidemiologic data for specific and effective management guidelines. Although PCR is a better diagnostic method, it uses a relatively small amount of blood from filter papers and could still underestimate *P*. *malariae* infections in samples with exceptionally low levels of parasitemia. More accurate prevalence data may be obtained from ultrasensitive PCR that targets multicopy regions of the parasite genome (*45*) or reverse transcription PCR of parasite RNA extracted from whole blood (*46*).

Sequences of the *csp* gene were shown to be highly polymorphic among *P. malariae* isolates from western Kenya. The most polymorphic region was in the central repeat region, where mutations and length differences were detected (*24**,**28*). Among the isolates from different geographic areas, *P. malariae* from East and Central/West Africa were genetically closely related and exhibited a comparable level of sequence variation. This variation could be attributed to positive selection, frequent recombination, and gene flow among the parasites, as follows. First, compared with *msp*1, *dhfr*, and *dhps* of *P. malariae *(*20**–**22*), the *csp* gene revealed a remarkably higher level of sequence diversity. It is possible that selection of *csp* genetic variants may confer immunogenic advantages to the pathogen during host invasion (*28**,**47*). Second, intense transmission and large vector populations in our study area might enhance frequent heterologous recombination of the parasite genome during reproduction in the mosquitoes and increase genetic diversity within populations (*24**,**25*). Third, recurrent gene flow between the parasite populations across countries, via human migration or dispersal of vector mosquitoes, promotes the spread of these genetic variants, leading to a lack of differentiation according to geographic region. Future study using other variable markers, such as microsatellites, on expanded population samples could validate our findings.

In summary, underestimation of the actual prevalence of asymptomatic infections hinders progress toward malaria elimination in Africa. The low parasitemia of *P. malariae* infections influences diagnostic sensitivity by microscopy. A more sensitive tool is needed to identify asymptomatic *P. malariae* and to improve control strategies, particularly among infants and children who are vulnerable to *P. malariae* infection.

Technical Appendix 1 Detailed description of PCR-based diagnostic assays and phylogenetic analyses of *Plasmodium* circumsporozoite protein gene sequences.

Technical Appendix 2 Additional information for *Plasmodium malariae *and *P. brasilianum* isolates.
