# Supplementary material for: Plasmodium malariae Prevalence and csp Gene Diversity, Kenya, 2014 and 2015
Source: Emerg Infect Dis. 2017 Apr;23(4):601–10. doi: 10.3201/eid2304.161245 (PMC5367407; doi:10.3201/eid2304.161245)
Supplement: Technical Appendix 1 — Detailed description of PCR-based diagnostic assays and phylogenetic analyses of Plasmodium circumsporozoite protein gene sequences. [file 16-1245-Techapp-s1.pdf]

Article DOI: http://dx.doi.org/10.3201/eid2304.161245

Plasmodium malariae Prevalence and *csp* Gene Diversity, Kenya, 2014 and 2015

Technical Appendix 1

Detailed description of PCR-based diagnostic assays and phylogenetic analyses of *csp* sequences

Microscopy

Slides were examined under microscopes 100× objective. Parasites were counted against 200 leukocytes.A slide was considered negative when no parasites were observed after counting over 100 microscopic fields. All slides were read in duplicate by two microscopists at the time of sample collection. In the case of discordance, the slides were examined by a third microscopist. The density of parasitemia was expressed as the number of asexual parasite per microliter of blood, assuming a leukocyte count of 8000 cells per microliter according to the WHO guidelines.

Nested PCR assay of Plasmodium species

Parasite DNA was extracted from half of a 50ul-dried blood spot by the Saponin/Chelex method (*1*). The final extracted volume was 200µl. A nested amplification of the 18S rRNA gene region of *Plasmodium (P. falciparum, P. vivax, P. malariae and P. ovale)* was used for parasite detection and species identification in all samples. DNA from *P. falciparum* isolates 7G8 (MR4-MRA-926) and HB3 (MR4-MRA-155), *P. vivax* Pakchong (MR4-MRA-342G) and Nicaragua (MR4-MRA-340G), *P. malariae* (MR4-MRA-179), as well as *P. ovale* (MR4-MRA-180) were used as positive controls in all amplifications. Water and uninfected samples were used as negative controls to ensure lack of contamination.Amplification was conducted in a 20ul reaction mixture containing 2ul of genomic DNA, 10ul of 2×DreamTaq™ Green PCR Master Mix (Fermentas), and 0.3uM primers. Reaction was performed in a BIORAD MyCycler thermal cycler following the published protocol (*2*). The amplified products were resolved electrophoretically on a 2% agarose gel in 0.5×Tris-borate (TBE) buffer and visualized under UV light.

Quantitative real-time PCR assay of Plasmodium species

Parasite DNA amount was estimated using the SYBR Green qPCR detection method with *Plasmodium*species-specific primers that targeted the 18S rRNA genes (*3*–*5*). Amplification was conducted in a 20µL reaction mixture containing 2µL of genomic DNA, 10µL 2×SYBR Green qPCR Master Mix (Thermo Scientific, USA), and 0.5µM primer. Reaction was performed in CFX96 Touch™ Real-Time PCR Detection System (Bio-Rad), with an initial denaturation at 95°C for 3 min, followed by 45 cycles at 94°C for 30 sec, 55°C for 30 sec, and 68°C for 1 min with a final 95°C for 10 sec. This was then followed by a melting curve step of temperature that ranged from 65°C to 95°C with 0.5°C increment to determine the melting temperature of each amplified product. Melting curve analyses were performed for each amplified sample to confirm specific amplifications of the target sequence. For the measure of reproducibility of the threshold cycle number (C_t), the mean value and standard deviations were calculated from triplicates in two independent assays.A cut-off threshold of 0.02 fluorescence units that robustly represented the threshold cycle at the log-linear phase of the amplification and above the background noise was set to determine *Ct* value for each assay. Samples yielding *Ct* values higher than 40 (as indicated in the negative controls) were considered negative for *Plasmodium* species.The parasite gene copy number (GCN) in a sample was quantified based on the threshold cycle using the follow equation (*6*): $GCN_{sample} = e^{[E \times \Delta Ct sample]}$, where GCN stands for gene copy number, ΔCt for the difference in threshold cycle between the negative control and the sample, and E for amplification efficiency. The amplification efficiency of primers was assessed on 10-fold serial dilutions ranging from 10^5 to 10^1 copies/µl of the control plasmids. DNA from *P. falciparum* isolates 7G8 (MR4-MRA-926) and HB3 (MR4-MRA-155), and *P. malariae* (MR4-MRA-179) isolate were used as positive controls. Water and uninfected samples were used as negative controls in all amplifications.

CSP sequencing and phylogenetic analyses

Four internal primers were designed specifically on the *P. malariaecsp* gene region and used together with the published primers (*7*) (Technical Appendix Table) to unambiguously amplify the three segments, the N-terminal, the central repeat, and the C-terminal regions of the *csp* gene. A total of 37 *P. malariae* isolates were amplified and sequenced. Amplification was conducted in a 20µL reaction mixture containing 2µL of genomic DNA, 10µL 2××DreamTaq™ Green PCR Master Mix (Fermentas) and 0.5µLof 10 µM primers. PCR cycles included an initial

denaturing step at 95°C for 3 min, 40 cycles of 95°C for 30 sec, 48-50°C for 30 sec, and 72°C for 2 min, followed by an additional extension at 72°C for 5 min in a Bio-Rad MyCycler Thermal Cycler. PCR products were visualized on 1% agarose gel and then purified and sequenced from both ends with BigDye Terminator v3.1 Cycle Sequencing Kit on an ABI 3130xl sequencer.

All obtained sequences were blasted against NCBI GenBank database for verification. They were translated into protein sequences and analyzed together with all available *csp* protein sequences of *P. malariae* as well as *P. brasilianum* retrieved from the GenBank database. Due to potential alignment errors associated with gaps in the nucleotide sequences, translated amino acid sequences with unambiguous indels were used in phylogenetic analyses. Sequences were aligned with MUSCLE v3.7 (*8*) using default settings followed by manual editing in Sequence Alignment Editor v1.d1 (*9*). A phylogenetic tree was reconstructed using the maximum likelihood method implemented in the PhyML program v3.0 (*10*). The WAG (Whelan And Goldman) substitution model,which assumes an estimated proportion of invariant sites and four gamma-distributed rate categories to account for rate heterogeneity across sites, was selected. Resulted trees were visualized in FigTree v1.4.2.

Sequence diversity including measures of evolutionary distances and average pairwise divergence were estimated using SSE v1.2 (*11*). The matrix-normalized distances based on the standard PAM model (*12*) that accounts for the probability of change from one amino acid to another were calculated. In addition, a similarity scan was performed between and within sequences using the standard PAM-Dayhoff matrix to normalize a matching score. Regions that meet or exceed the set criteria of the number of matches were plotted on a dot plot graph. *Plasmodium malariae* of East Africa (Kenya and Uganda), Central/West Africa (Cameroon and Cote d'Ivoire), and South America (Brazil and Venezuela) were compared for level of sequence diversity among gene regions.

References

1. Wooden J, Kyes S, Sibley CH. PCR and strain identification in *Plasmodium falciparum.* Parasitol Today. 1993;9:303–5. PubMed http://dx.doi.org/10.1016/0169-4758(93)90131-X

2. Kimura K, Kaneko O, Liu Q, Zhou M, Kawamoto F, Wataya Y, et al. Identification of the four species of human malaria parasites by nested PCR that targets variant sequences in the small subunit rRNA gene. Parasitol Int. 1997;46:91–5. http://dx.doi.org/10.1016/S1383-5769(97)00013-5

3. Rougemont M, Van Saanen M, Sahli R, Hinrikson HP, Bille J, Jaton K. Detection of four *Plasmodium* species in blood from humans by 18S rRNA gene subunit-based and species-specific real-time PCR assays. J Clin Microbiol. 2004;42:5636–43. PubMed http://dx.doi.org/10.1128/JCM.42.12.5636-5643.2004

4. Phuong M, Lau R, Ralevski F, Boggild AK. Sequence-based optimization of a quantitative real-time PCR assay for detection of *Plasmodium ovale* and *Plasmodium malariae.* J Clin Microbiol. 2014;52:1068–73. PubMed http://dx.doi.org/10.1128/JCM.03477-13

5. Lo E, Nguyen J, Oo W, Hemming-Schroeder E, Zhou G, Yang Z, et al. Examining *Plasmodium falciparum* and *P. vivax* clearance subsequent to antimalarial drug treatment in the Myanmar-China border area based on quantitative real-time polymerase chain reaction. BMC Infect Dis. 2016;16:154–66. PubMed http://dx.doi.org/10.1186/s12879-016-1482-6

6. Lo E, Zhou G, Oo W, Afrane Y, Githeko A, Yan G. Low parasitemia in submicroscopic infections significantly impacts malaria diagnostic sensitivity in the highlands of Western Kenya. PLoS One. 2015;10:e0121763. PubMed http://dx.doi.org/10.1371/journal.pone.0121763

7. Tahar R, Ringwald P, Basco LK. Heterogeneity in the circumsporozoite protein gene of *Plasmodium malariae* isolates from sub-Saharan Africa. Mol Biochem Parasitol. 1998;92:71–8. PubMed http://dx.doi.org/10.1016/S0166-6851(97)00226-0

8. Edgar RC. MUSCLE: multiple sequence alignment with high accuracy and high throughput. Nucleic Acids Res. 2004;32:1792–7. PubMed http://dx.doi.org/10.1093/nar/gkh340

9. Rambaut A. Se-Al: sequences alignment editor v. 2.0a11. Edinburgh: Institute of Evolutionary Biology. 2002 [cited 2016 Jul 1]. http://tree.bio.ed.ac.uk/software/

10. Guindon S, Dufayard JF, Lefort V, Anisimova M, Hordijk W, Gascuel O. New algorithms and methods to estimate maximum-likelihood phylogenies: assessing the performance of PhyML 3.0. Syst Biol. 2010;59:307–21. PubMed http://dx.doi.org/10.1093/sysbio/syq010

11. Simmonds P. SSE: a nucleotide and amino acid sequence analysis platform. BMC Res Notes. 2012;5:50–9. PubMed http://dx.doi.org/10.1186/1756-0500-5-50

12. Dayhoff MO, Schwartz RM, Orcutt BC. A model of evolutionary change in proteins. In: Dayhoff MO, editor. Atlas of protein sequence and structure. Vol. 5, Suppl. 3. Washington: National Biomedical Research Foundation; 1979. p. 345–52

Technical Appendix Table. Primer sequences and PCR conditions of the circumsporozoite protein (*csp*) gene

Primer name	Sequence	Expected size (bp)	Annealing temperature
csp-F*	ATGAAGAAGTTATCTGTCTTAGCAATATCC	280	50°C
csp-280R	CCGGGGGGTTGTTTCAATTTATTTTC		
csp-280F	GCTGTTGAAAATAAATTGAAACAACC	700-800	48°C
csp-1070R	CCACTTTATTATCCTTATTTTTCGC		
csp-1070F	GCGAAAAATAAGGATAATAAAGTGG	400	50°C
csp-R*	TTAGTGAAAGAGTATTAAGACTAAAAC		

***Primer published in (*7*).

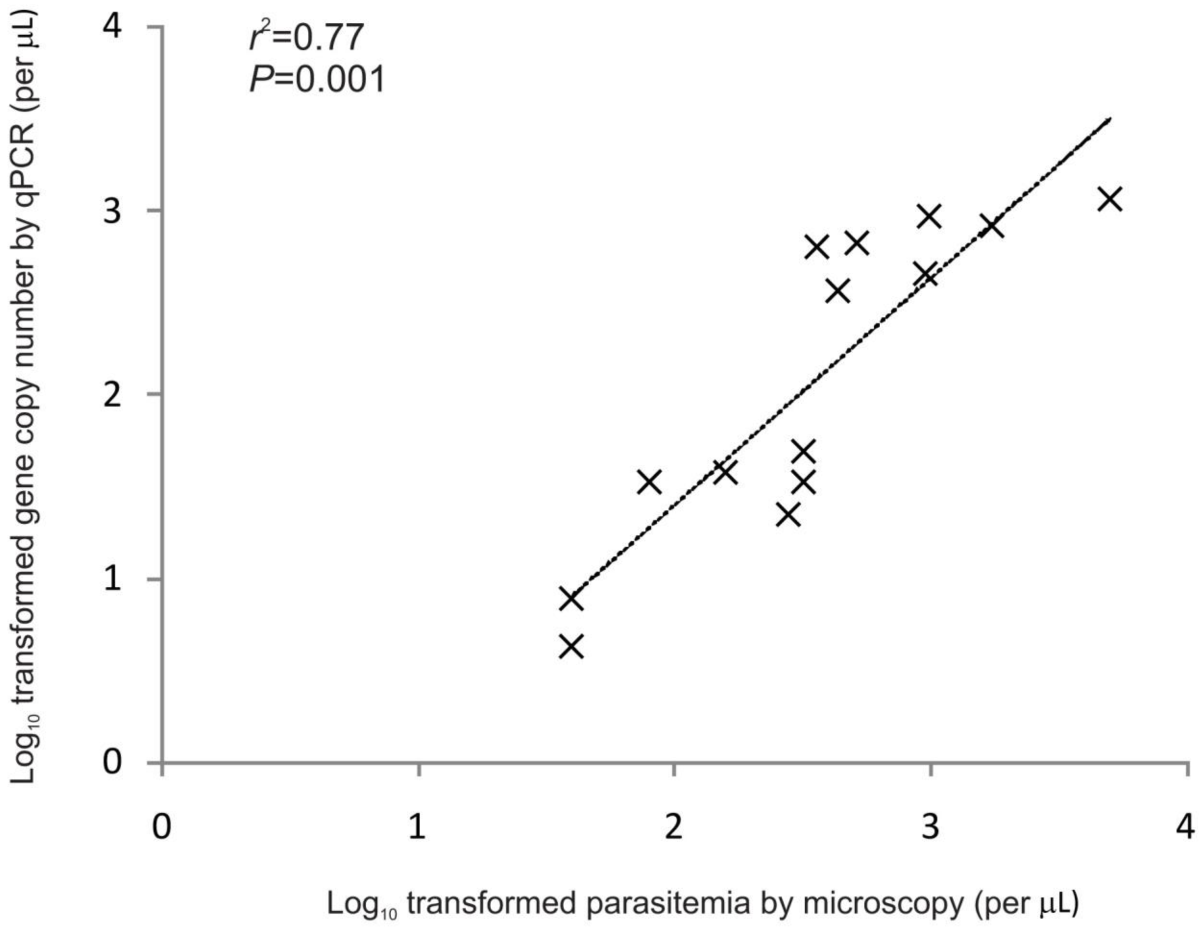

Technical Appendix Figure 1. Scatter plot showing the significant correlation of parasite gene copy number measured by quantitative real-time PCR and parasitemia by microscopy of *Plasmodium malariae* isolates.

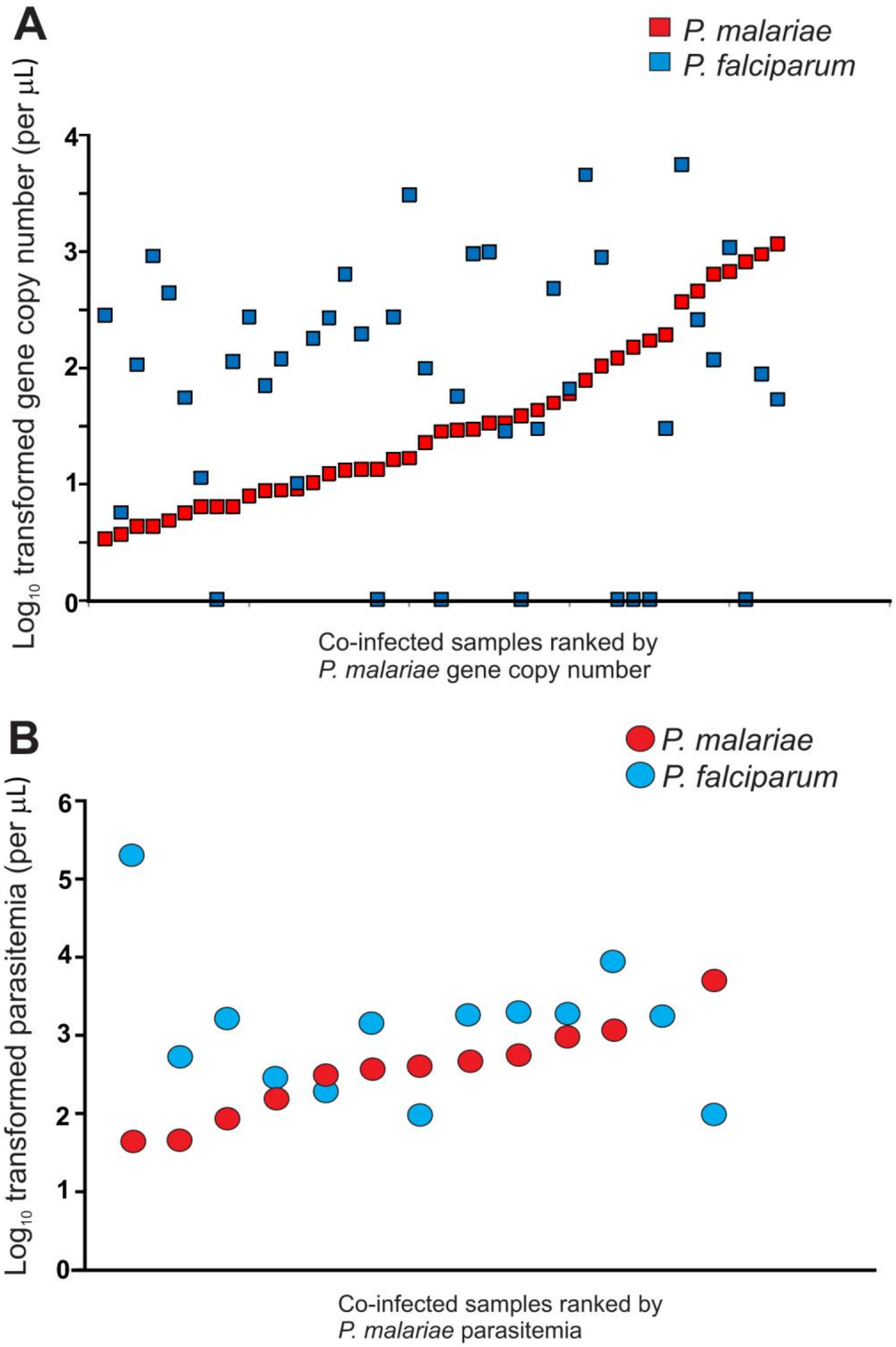

Technical Appendix Figure 2. Scatter plots showing (A) parasite gene copy number of *P. malariae* and *P. falciparum* ranked from low to high P. *malariae* parasite gene copy number and (B) parasitemia of *P. malariae* and *P. falciparum* ranked from low to high *P. malariae* parasitemia of co-infected samples.

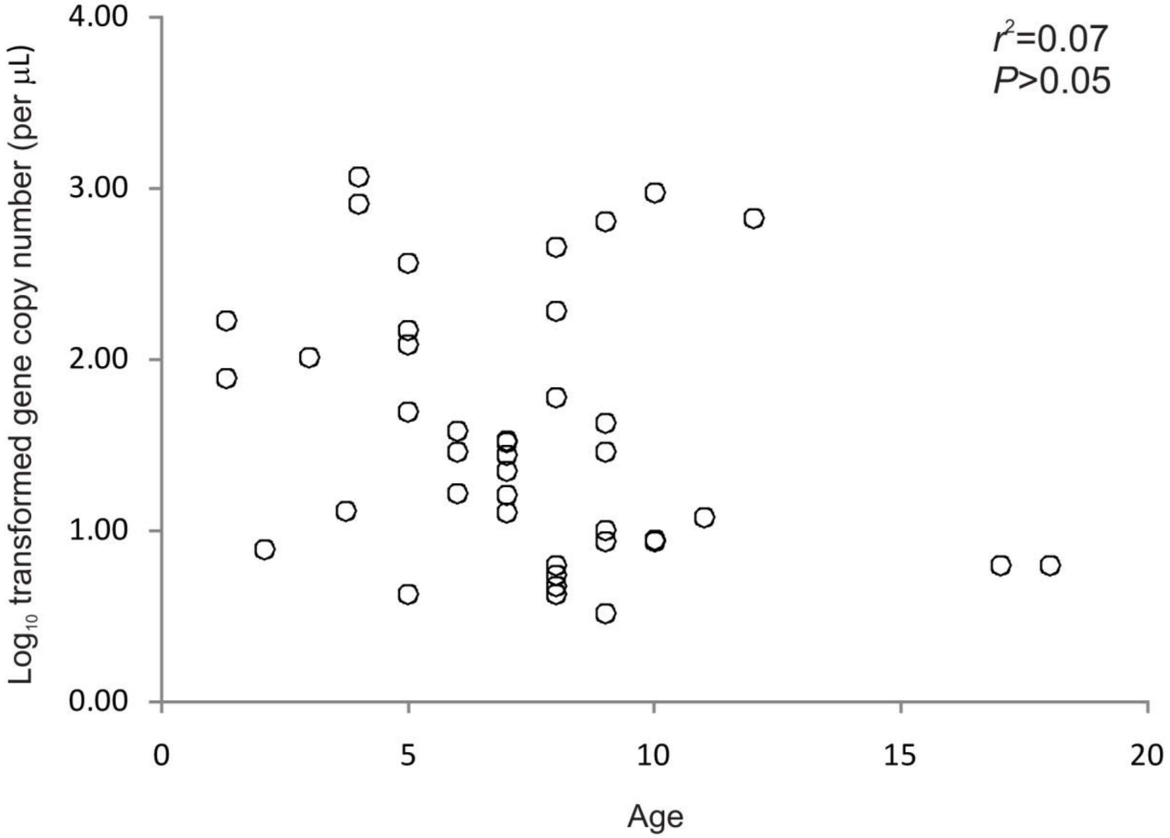

Technical Appendix Figure 3. Scatter plot showing the non-significant correlation of *P. malariae* gene copy number against age among samples.
